# Automated Audit and Self-Correction Algorithm for Seg-Hallucination Using MeshCNN-Based On-Demand Generative AI

**DOI:** 10.3390/bioengineering12010081

**Published:** 2025-01-16

**Authors:** Sihwan Kim, Changmin Park, Gwanghyeon Jeon, Seohee Kim, Jong Hyo Kim

**Affiliations:** 1 Department of Applied Bioengineering, Graduate School of Convergence Science and Technology, Seoul National University, Seoul 08826, Republic of Korea; imksh0707@snu.ac.kr (S.K.); smilecmp@snu.ac.kr (C.P.); ksh0529@snu.ac.kr (S.K.); 2 ClariPi Research, ClariPi Inc., Seoul 03088, Republic of Korea; khjeon3014@claripi.com; 3 Department of Radiology, Seoul National University Hospital, Seoul 03080, Republic of Korea; 4 Department of Radiology, Seoul National University College of Medicine, Seoul 03080, Republic of Korea

**Keywords:** AI audit, Seg-Hallucination, uncertainty, anomaly screening, segmentation

## Abstract

Recent advancements in deep learning have significantly improved medical image segmentation. However, the generalization performance and potential risks of data-driven models remain insufficiently validated. Specifically, unrealistic segmentation predictions deviating from actual anatomical structures, known as a Seg-Hallucination, often occur in deep learning-based models. The Seg-Hallucinations can result in erroneous quantitative analyses and distort critical imaging biomarker information, yet effective audits or corrections to address these issues are rare. Therefore, we propose an automated Seg-Hallucination surveillance and correction (ASHSC) algorithm utilizing only 3D organ mask information derived from CT images without reliance on the ground truth. Two publicly available datasets were used in developing the ASHSC algorithm: 280 CT scans from the TotalSegmentator dataset for training and 274 CT scans from the Cancer Imaging Archive (TCIA) dataset for performance evaluation. The ASHSC algorithm utilizes a two-stage on-demand strategy with mesh-based convolutional neural networks and generative artificial intelligence. The segmentation quality level (SQ-level)-based surveillance stage was evaluated using the area under the receiver operating curve, sensitivity, specificity, and positive predictive value. The on-demand correction performance of the algorithm was assessed using similarity metrics: volumetric Dice score, volume error percentage, average surface distance, and Hausdorff distance. Average performance of the surveillance stage resulted in an AUROC of 0.94 ± 0.01, sensitivity of 0.82 ± 0.03, specificity of 0.90 ± 0.01, and PPV of 0.92 ± 0.01 for test dataset. After the on-demand refinement of the correction stage, all the four similarity metrics were improved compared to a single use of the AI-segmentation model. This study not only enhances the efficiency and reliability of handling the Seg-Hallucination but also eliminates the reliance on ground truth. The ASHSC algorithm offers intuitive 3D guidance for uncertainty regions, while maintaining manageable computational complexity. The SQ-level-based on-demand correction strategy adaptively minimizes uncertainties inherent in deep-learning-based organ masks and advances automated auditing and correction methodologies.

## 1. Introduction

Medical image segmentation is a fundamental task in medical image analysis, enabling the precise delineation of anatomical structures and pathological regions. U-Net, introduced by Ronneberger et al., has become a widely adopted architecture for 2D medical image segmentation tasks. Its symmetric encoder–decoder structure with skip connections allows the model to leverage low-level and high-level features, achieving remarkable performance across various medical imaging modalities, including CT, MRI, and X-rays [1,2].

Despite its success, U-Net operates on individual 2D slices of medical images, limiting its ability to capture spatial continuity across slices in 3D volumetric data. This limitation becomes significant when analyzing imaging modalities such as CT or MRI, where the spatial relationship between slices is crucial for accurate segmentation [1,3].

To address this challenge, Çiçek et al. extended the U-Net architecture to volumetric data by introducing 3D U-Net. The 3D U-Net replaces 2D convolutional and pooling operations with 3D counterparts, enabling the model to process 3D medical imaging volumes holistically. This advancement allows for better modeling of spatial dependencies, leading to more consistent and accurate segmentation results for complex anatomical structures [3,4].

Recent advances in 3D-based medical image segmentation have largely centered on Transformer-based, CNN-based, and hybrid approaches. Transformer-based models like VISTA3D, which supports automatic (up to 127 classes) and interactive segmentation, and SegFormer3D, which uses a hierarchical Transformer and all-MLP decoder, demonstrate strong performance with fewer parameters and reduced computational overhead [5,6]. Meanwhile, CNN-based models, exemplified by nnU-Net, remain robust and widely adopted despite their large parameter counts [7,8]. Finally, hybrid approaches such as MedSAM, trained on over 1.5 million image-mask pairs across 10 modalities, combine the strengths of different architectures to offer high accuracy and broad applicability [9].

In addition, generative AI approaches are emerging as transformative solutions to address limitations in data availability and domain generalization for medical image segmentation. Techniques such as GenSeg integrate generative models to synthesize high-fidelity image–mask pairs, enabling robust performance in ultra-low-data regimes by optimizing data generation in alignment with segmentation needs [10]. Generative medical segmentation (GMS), similarly, leverages pre-trained vision foundation models to create latent representations for segmentation tasks, significantly reducing trainable parameters while enhancing generalization across domains. GMS has demonstrated superior performance across multiple datasets and modalities, showcasing its potential to reduce dependency on large annotated datasets [11]. By bridging data generation and segmentation through multi-level optimization frameworks (e.g., GenSeg) or latent mapping techniques (e.g., GMS), generative approaches provide scalable and efficient alternatives to traditional methods, particularly in scenarios with limited labeled data [10,11]. These innovations pave the way for broader adoption of AI in medical image segmentation.

Despite the emergence and development of the state-of-the-art (SOTA) models in the medical image segmentation, erroneous results have been consistently reported across various SOTA models. Ma et al. observed that the MedSAM model struggled with weak boundaries and low-contrast regions, resulting in over-segmentation or under-segmentation, especially when user inputs are ambiguous or incomplete [9]. Gunawardhana et al. analyzed that the nnU-Net model could experience performance degradation when dealing with complex structures or domain shifts, particularly when the data deviates from the training distribution, leading to segmentation errors [12]. He et al. highlight challenges in medical image segmentation, including unclear tumor boundaries, the limitations of single-point inputs, and the localized nature of super voxel representations, leading to conservative predictions and difficulties in domain generalization [5]. These studies have highlighted remaining challenges in deep-learning-based segmentation within medical imaging, emphasizing its complexity and the need for further advancements.

In the context of medical imaging, a segmentation hallucination (Seg-Hallucination) refers to the generation of false or misleading features by a deep-learning-based model during the image segmentation process. This phenomenon occurs when the model predicts structures or boundaries that do not exist in the actual imaging data. Such hallucinations can be introduced due to various factors, including noise in the data, deformable tissue structures, disease-related pathologies, or biases within the training dataset.

Early investigations into the Seg-Hallucination have demonstrated its frequent occurrence in data-driven neural networks used for an image segmentation workflow [13,14,15,16,17,18]. These studies identified several underlying causes of the Seg-Hallucination, including model overfitting, inadequate training datasets, and the incorporation of noise or artifacts within imaging data. For example, Zhang et al. examined the effects of dataset diversity on Seg-Hallucination prevalence, revealing that models trained on heterogeneous datasets showed a marked reduction in the hallucination rates compared to those trained on narrower data distributions [13].

In terms of clinical impact, Rickmann and collogues advocated the clinically relevant problem of the hallucination on the organ segmentation [14]. Their findings indicated that the Seg-Hallucination significantly affected diagnostic reliability, with a potential increase in false-positive rates during segmentation tasks. Furthermore, research conducted by Biase et al. highlighted the downstream effects of hallucinated segments on radiation treatment pathways, underscoring the potential for erroneous clinical decisions based on faulty segmentation outputs [15].

To enhance the robustness of the DL-based segmentation model, recent studies have proposed various mitigation strategies. For example, Ma et al. suggested the implementation of adversarial training approaches to secure model robustness against erroneous predictions in the medical image segmentation [16]. However, addressing the Seg-Hallucination requires careful consideration of the model techniques such as uncertainty quantification to ensure that deep-learning-based predictions are reliable and accurate. Accordingly, Nair et al. advocated for integrating uncertainty quantification methods in segmentation pipelines, enabling clinicians to assess the reliability of model outputs before making crucial medical decisions [19].

Despite the growing efforts to comprehend the mechanisms and influences of seg-hallucination and to develop effective solutions, the task remains challenging. The future solution could encourage continued interdisciplinary collaboration to refine segmentation algorithms and enhance their reliability in clinical settings, ultimately improving patient care outcomes.

As deep-learning-based techniques continue to be integrated into clinical settings for applications such as tumor detection, organ delineation, and anomaly recognition, understanding and addressing the Seg-Hallucination has become imperative. However, most prior studies have focused on investigating underlying mechanisms and assessing clinical implications. The objectives of this research on the Seg-Hallucination extend beyond mere identification of the phenomenon. By developing practical mitigation strategies for the Seg-Hallucination, enhancing trust and contributing to the scientific discourse, we aspire to address a critical challenge in the field of medical imaging. Therefore, in this study, we propose the automated Seg-Hallucination surveillance and correction (ASHSC) algorithm that can enhance the reliability of segmentation outputs, applying 3D segmentation information derived from CT images without relying on predefined ground-truth label. The insights gained from this study will not only advance our understanding of the Seg-Hallucination but also promote the safe and effective integration of deep-learning-based segmentation technologies into clinical practice. The key contributions are as follows:

▪ We proposed a novel fully automated audit and self-correction algorithm handling the Seg-Hallucination, utilizing the MeshCNN and the generative AI.
▪ Differentiated from prior studies, surveilling deformities in 3D surface topology, the ASHSC algorithm eliminates reliance on the ground truth and allows an intuitive guide for uncertainty regions.
▪ We newly formulated the segmentation quality level (SQ-level) based on the ratio of uncertainty faces measured in the 3D-mesh surface.
▪ Even in correcting the Seg-Hallucination, we effectively minimized unnecessary correction tasks through the SQ-level-based on-demand strategy.
▪ By employing binary masks exclusively for surveilling and correcting the Seg-Hallucination, we present an algorithm characterized by manageable computational complexity, thereby ensuring its practical application across diverse testing environments within the hospital setting.

## 2. Materials and Methods

### 2.1. Datasets

**Figure 1:**
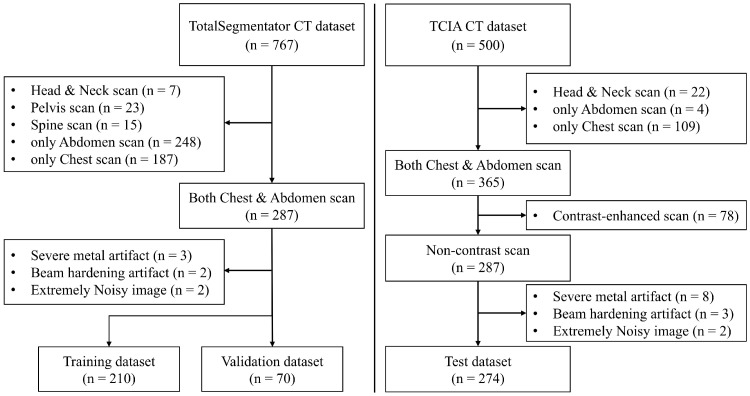
Flow diagram illustrating the case selection and inclusion criteria for data refinement yielding the training, validation, and test datasets.

This study used a CT dataset collected from open-public storages provided by the TotalSegmentator and the Cancer Imaging Archive (TCIA) [20,21]. The exclusion criteria were as follows: (1) chest and abdominal regions are not captured simultaneously; (2) intravenous contrast-injected CT scan; (3) severe metal artifact; (4) beam hardening artifact; and (5) extremely noisy image. Finally, a total of 554 patients were enrolled for the experiments (Figure 1). The TotalSegmentator CT dataset (n = 280) was used for training (n = 210) and validation (n = 70) of the ASHSC algorithm, while performance testing of the algorithm was conducted using the TCIA CT dataset (n = 274). The TCIA dataset, employed for reliable evaluation across a variety of data, composed CT images from both diseased and normal patients. The final inclusion CT datasets were used for extracting a specific organ (e.g., heart) mask utilizing AI-based segmentation (AI-seg) SW.

**Figure 2:**
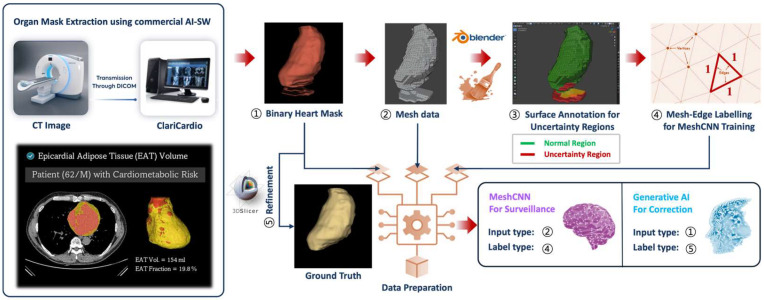
Overall schematics for the experimental data preparation procedure.

For the purpose-specific data preparation for training and evaluation of the ASHSC algorithm, a commercial and two open-source software programs were used (Figure 2). Firstly, to train and evaluate the suggested algorithm handling the Seg-Hallucination, binary mask of the heart was obtained for inclusion datasets using dedicated AI-based software (ver. 1.0, ClariCardio, ClariPi Inc., Seoul, Republic of Korea, https://claripi.com/claricardio, accessed on 1 December 2024). After an extraction of binary heart mask, technologists modified the prediction masks aligning with ideal organ shape to generate a paired AI-predicted organ mask and the ground truth. The modification software was an opensource software (ver. 5.6.2, 3D Slicer, Boston, MA, USA, http://www.slicer.org, accessed on 3 December 2024) [22]. Secondly, for preparation of the training data in the surveillance model, open-source mesh-handling software (ver. 4.3, blender, Blender Foundation, Amsterdam, The Netherlands, https://www.blender.org, accessed on 3 December 2024) was utilized. The binary masks were transformed into mesh-type data, and it underwent an annotation of the poorly segmented surface areas identified as uncertainty regions. As an additional task for deep learning training, the individual edges within the mesh data obtained from the Blender software were labeled as either normal regions or uncertainty regions, each as a separate learning class. For the correction model training, volumetric organ masks of the training 210 cases were redefined only for the normal organ regions, excluding the uncertainty regions annotated in the training data of surveillance model.

### 2.2. Overall Procedure

**Figure 3:**
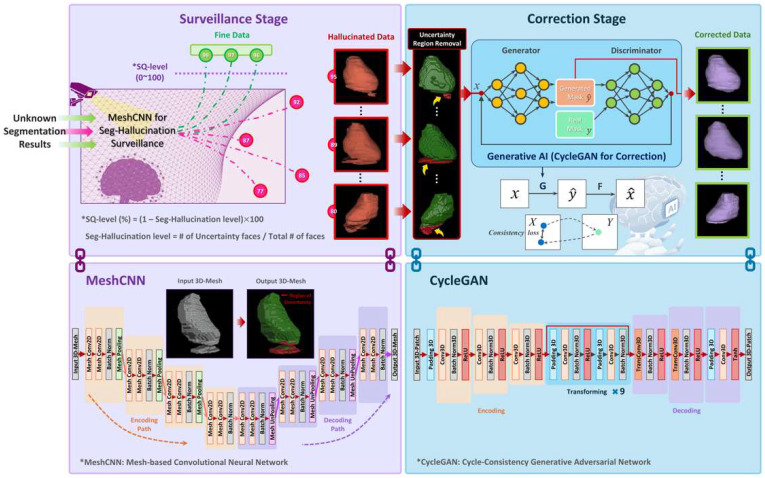
A schematic overview of the ASHSC algorithm pipeline, comprising a MeshCNN-based surveillance stage to detect and quantify the Seg-Hallucination, followed by a correction stage that employs an on-demand generative AI model (CycleGAN) to refine the uncertainty regions.

In this study, automated anti-hallucination algorithm on CT image segmentation was proposed based on two-stage deep learning-based architecture. The prior stage involved the surveillance of the Seg-Hallucination, while the subsequent stage focused on minimizing the uncertainty region and correcting the Seg-Hallucination to align with normal anatomical structures (Figure 3).

All the data processing was performed on a workstation (Intel Core i9-9900k, 128 GB RAM, and NVIDIA GeForce RTX 3090). The deep learning framework was PyTorch (ver. 2.5.1), and the experiments were conducted in the python (ver. 3.11.9) environment.

### 2.3. Automated Seg-Hallucination Surveillance Stage

To extract the imaging features for identifying an uncertainty surface region of the segmented object, a convolutional neural network (CNN) was used. As a goal of the surveillance model is capturing an irregular representation of 3D object shape, a polygonal mesh-based CNN (MeshCNN) was used to explicitly handle both shape surface and topology [23]. Analogous to classic CNNs, the MeshCNN combines convolution and pooling layers specially designed for mesh edges. By generating new mesh connectivity for the subsequent edge-based convolutions and pooling procedure, the MeshCNN keeps intrinsic geodesic connections within the object mesh. Throughout iterative edge-based CNN operations, the DL model learned how to disregard the redundant information and remain minimal key features. The MeshCNN model used a U-shaped encoding–decoding structure as the backbone. It takes 3D mesh data as input starting with 32 channels and sequentially undergoes four down-sampling depths to extract features. It then performs symmetrical up-sampling to produce 3D mesh data as the output, maintaining the same dimensionality as the input mesh data. To set the pooling resolution for the down-sampling and up-sampling, this study determined the maximum number of edges measured within the mesh dataset used for training and validation. The task-based mesh pooling resolution values in four-depth network architecture were then sequentially set to (11,000, 9000, 6000, 3000). Exceptionally, when the number of edges for specific input mesh exceeded the preset value of first-pooling layer, the first-pooling layer resolution was adaptively modified to handle each outlier case.

When training the MeshCNN model, the data preprocessing pipeline involved multiple steps. Data resizing performed transition of organ binary mask from anisotropic volumetric dimension to isometric voxel dimension with uni-spacing (1 × 1 × 1 mm^3^). The isometric volume mask was transformed to the mesh-type data, including vertices, faces, and edges. Data cleaning removed invalid mesh faces (zero-area faces or non-manifold faces), identifying valid edge features for the model training. Geometric features like dihedral angle, two opposite angles, and two length ratios are computed for an individual edge to form a 5-dimensional edge-feature vector as the input of the MeshCNN model. Data augmentation included a vertex coordinate scaling with Gaussian filter, a mesh-edge flipping, and sliding vertices along with neighboring edges to adjust subtle mesh variations. After the data augmentation, edge-feature input vectors were standardized with mean and standard deviation of entire edge-feature vectors in training data. This systematic preprocessing cleaned, augmented, and standardized the data for effective neural network training.

The network was optimized by an Adam optimizer with an initial learning rate of 0.0001 and a beta1 value of 0.9. The learning rate scheduler used lambda, where the learning rate remained constant for the first half of the epochs and was gradually decreased to zero for the remaining half of the epochs. The loss function used for the mesh segmentation was weighted cross-entropy ($L_{WCE}$), with the ratio (*α* = 0.1) of the normal class to the uncertain class set at 1:9 (Equation (1)). To overcome the limitations of the limited number of training data, anisotropic scaling of vertex locations was applied as an augmentation method to enhance the generalization performance of the MeshCNN model. Through the augmentation methodology, the number of training data for MeshCNN was increased by approximately 20 times (4200 mesh objects), with each datum being randomly shuffled for training purposes.

(1) $$L_{WCE}=-\alpha ylog\left(\hat{y}\right)-\left(1-y\right)\log\left(1-\hat{y}\right)$$

(*α* denotes scaling parameter penalizing false positives or false negatives more harshly).

As the mesh explicitly represents the topology of an object surface, we applied it for automated annotation to mark the uncertainty surface regions of 3D organ masks. After the initial prediction of uncertain surface edges, the uncertainty regions with smaller than pre-set threshold value of 12 mm^2^ were converted to normal regions to reduce the false positives. When the predicted uncertainty surfaces were determined to be the Seg-Hallucination, they were redefined as a closed surface segments using the mesh-based convex-hull technique. Sequentially, the triangular meshes of uncertainty surface were transformed into a 3D voxel grid representation with the k-d tree nearest neighbor interpolation (KDT) algorithm. Finally, they were subtracted from volumetric input data to eliminate the uncertainty regions.

### 2.4. Segmentation Quality Level and Optimal Threshold Justification

In the procedure of predicting the uncertainty surface regions in MeshCNN model, we novelly devised a metric to quantitatively measure the segmentation quality level (SQ-level). The SQ-level was formulated with a following equation, and a Seg-Hallucination level was defined as the number of uncertainty faces to the total number of mesh faces (Equation (2)). The SQ-level ranged from 0 to 100 with a percentage scale and was designed to serve as a threshold for distinguishing the Seg-Hallucination data.

(2) $$∴SQ-level=\left(1-SegHallucination\;level\right)\times 100=\left(1-\frac{The\;number\;of\;uncertainty\;mesh\;faces}{The\;number\;of\;total\;mesh\;faces\;}\right)\times 100$$

The optimal SQ-level was determined using the receiver operating characteristic (ROC) curve obtained from a blinded observer study identifying the Seg-Hallucination in AI segmentation results impacting future quantitative analyses. It was identified as the point maximizing the sensitivity while minimizing the false positive rate. It is typically achieved by locating the point on the curve closest to the upper left corner, representing the ideal scenario of achieving high sensitivity and specificity simultaneously. Justification for the chosen SQ-level is based on its ability to balance the trade-offs between sensitivity and specificity, thereby providing a clinically relevant criterion that optimally distinguishes the Seg-Hallucinations and normal cases. In addition, the optimal SQ-level selection may consider the context of the task-specific application, including the consequences of false positives versus false negatives in order to ensure that the SQ-level based MeshCNN model performs effectively in clinical scenarios.

### 2.5. On-Demand Correction Stage for Seg-Hallucination

To prevent unnecessary corrections for normal region of the target object, below the preset SQ-level threshold, the uncertainty regions were localized and adaptively removed. As the surface of the remaining area could be rough and inaccurate, post-processing based on a generative AI (GenAI) model was performed to retain the skeletal structure of the organ while achieving a smooth and natural style similar to the ground truth. The AI-seg results were preserved to the greatest extent in areas where the initial AI-seg model performed effectively, while a refinement work was conducted using a GenAI-based on-demand approach solely in regions characterized by high uncertainty. In this study, we called this adaptive strategy an on-demand correction. Among the numerous GenAI models, a 3D-based cycle-consistency generative adversarial network (CycleGAN) was employed for its strength on maintaining an identity structure in three-dimensional space [24]. The network was composed of four main components with two generators (G and F) and two discriminators ($D_{Y}$ and $D_{X}$). The ResNet-based generator contained three main parts: encoder, transformer, and decoder. The encoder block extracts the latent vectors through the down-sampling, and the nine-times-repeated transformer performed the mapping of the targeted style to the latent vectors. Finally, the decoder up-sampled the condensed features to original input shapes, trying to maintain the input object structures. The network received a volume patch (64 × 64 × 64)-shaped binary organ mask as the input data and produced a volume patch of the same dimensions, styled according to the target ground truth. All output volume patches were stacked and finally stitched to a single volumetric datum.

For preprocessing the training data, the volumetric binary mask was resized to an isometric shape with uni-spacing voxel dimension (1 × 1 × 1 mm^3^). After the transformation, 64 × 64 × 64 volume patches were generated with a random cropping. In the random cropping, a threshold for the number of non-zero voxels was set based on 0.1% of the total number of volume patches (approximately 262 voxels). The volume patches above the threshold were included for the AI model training. Nevertheless, to minimize the class imbalance problem, ten percent of low-information volume patches were retained for training. For the efficient deep learning training, the intensity value range of all volume patches was normalized as the range of 0 to 1.

The network was optimized with the Adam optimizer with an initial learning rate of 0.002 and a beta1 value of 0.9. The learning rate scheduler used lambda, and the training dataset was randomly shuffled. To prevent an infinite number of generating scenarios where the discriminator could be confused, three types of object functions were concurrently used as the total loss ($L_{Total}$) with (Equation (3)): one for generative adversarial network loss ($L_{GAN}$), another for cycle-consistency loss ($L_{CYC}$), and the other for identity loss ($L_{IDEN}$) (Equations (4)–(6)). In total loss, the $\lambda_{c}$ and $\lambda_{i}$ were set as 10 and 0.5, respectively.

(3) $$L_{Total}\left(G,F,\;D_{X},\;D_{Y}\right)=L_{GAN}\left(G,D_{Y},X,Y\right)+L_{GAN}\left(F,D_{X},Y,X\right)+\lambda_{c}L_{CYC}\left(G,F\right)+\lambda_{c}\lambda_{i}L_{IDEN}\left(G,F\right)$$

(4) $$L_{GAN}\left(G,D_{Y},X,Y\right)=E_{y\sim p_{data}\left(y\right)}\left[logD_{Y}\left(y\right)\right]+E_{x\sim p_{data}\left(x\right)}\left[log(1-D_{Y}\left(G(x\right)\right]$$

(5) $$L_{CYC}\left(G,F\right)=E_{x\sim p_{data}\left(x\right)}\left[{\left\|F\left(G(x)\right)-x\right\|}_{1}\right]+E_{y\sim p_{data}\left(y\right)}\left[{\left\|G\left(F(y)\right)-y\right\|}_{1}\right]$$

(6) $$L_{IDEN}\left(G,F\right)=E_{x\sim p_{data}\left(x\right)}\left[{\left\|F\left(x\right)-x\right\|}_{1}\right]+E_{y\sim p_{data}\left(y\right)}\left[{\left\|G\left(y\right)-y\right\|}_{1}\right]\;$$

### 2.6. Statistical Analysis

In the evaluation of the surveillance model, sensitivity, specificity, positive predictive value (PPV), and area under the receiver operating curve (AUROC) were employed as metrics [24,25]. In the AUROC analysis, three technologists conducted a blinded observer study to assess the performance of the SQ-level-based seg-hallucination surveillance, utilizing a binary classified answer sheet to ascertain whether the AI-seg result contained the Seg-Hallucination impacting on future quantitative assessments. All diagnostic analyses were conducted using SPSS (version 25.0; IBM Corp., Armonk, NY, USA).

For the correction model evaluation, volumetric Dice score, volume error percentage, average surface distance (ASD), and Hausdorff distance (HD) were used as the metrics [26,27,28,29]. The continuous variables were presented as mean ± standard deviation. Statistical significance was defined as a *p*-value < 0.05.

For evaluating feasibility for real-time or clinical use, computational complexity of the ASHSC algorithm was analyzed on five different testing environments in a clinical setting.

## 3. Results

Seventy validation cases and two hundred and seventy-four test cases were employed for a performance evaluation. The mean SQ-levels (%) of the two evaluation datasets were shown as 95.2 ± 8 and 93.8 ± 5.

### 3.1. SQ-Level-Based Surveillance Stage Algorithm

In blinded-observer studies, the average optimal SQ-level thresholds from the three observers were 97.6% and 95.3% for the validation and test dataset, respectively (Table 1). Table 1 shows the surveillance performance of surveillance stage using the SQ-level-based MeshCNN model over the evaluation datasets. Based on the ground truth of three observers, the mean AUROC values were 0.93 ± 0.02 and 0.94 ± 0.01 in the validation and test dataset, respectively. At the optimal threshold of SQ-level identified within each ROC curve, sensitivity, specificity, and positive predictive value (PPV) were demonstrated as 0.85 ± 0.04 vs. 0.82 ± 0.03, 0.85 ± 0.01 vs. 0.90 ± 0.01, and 0.84 ± 0.02 vs. 0.92 ± 0.01 in the validation and test datasets, respectively.

### 3.2. Performance of the ASHSC Algorithm

**Figure 4:**
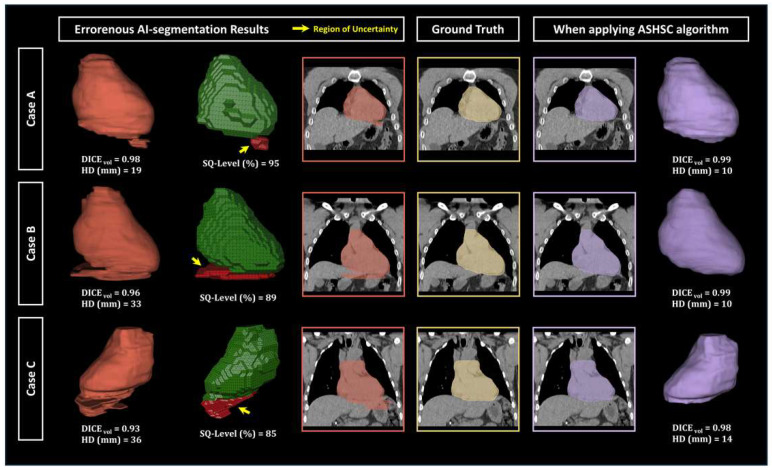
Segmentation outcomes for three representative cases (A–C) across different SQ-levels. Each row displays the initial AI-generated segmentation with its SQ-level and associated similarity scores (left), the ground truth segmentation (middle), and the final segmentation obtained after applying the ASHSC algorithm (right). The displayed similarity metrics include volumetric Dice score (DICE_vol_) and Hausdorff distance (HD). The yellow arrow indicates the region of uncertainty.

Figure 4 shows examples of the applicating the ASHSC algorithm on three representative cases with different SQ-levels. After the application of the ASHSC algorithm, in a perspective of qualitative evaluation, the uncertainty regions related to the Seg-Hallucination were dominantly removed on each erroneous result. From the perspective of quantitative evaluation, it was confirmed that the volumetric dice score and the Harsdorf distance were significantly improved compared to the case of using a single AI-based segmentation (AI-seg) model.

**Figure 5:**
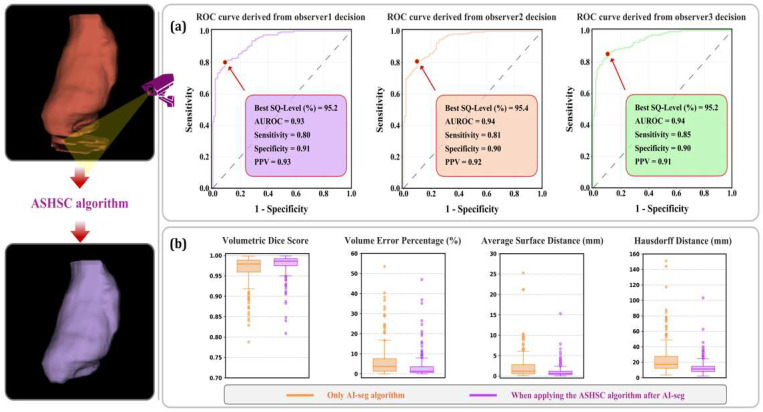
Performance of the ASHSC algorithm on the test dataset: (**a**) SQ-level-based surveillance performance; (**b**) improved performance when applying the proposed algorithm after AI-based segmentation.

Figure 5 illustrates the performance of the surveillance and correction stages within the proposed algorithm as applied to the test dataset. When the MeshCNN with on-demand generative AI (GenAI) was applied using the best mean SQ-level (95.3%), it was confirmed that the performance of the algorithm was improved for all four similarity metrics.

### 3.3. Performance Comparisons Across Problem-Solving Methodologies

Beyond the end-to-end performance evaluation, to evaluate how the ASHSC algorithm performs on the AI-seg results from different problem-solving methodologies, we compared three methodologies (AI-seg with MeshCNN vs. AI-seg with GenAI vs. AI-seg with both MeshCNN and GenAI) with respect to the single usage of the AI-seg. For both the validation and test datasets, the analysis indicated that the highest performance was attained when integrating both MeshCNN and generative AI into the AI-seg model (Table 2). The use of only GenAI with the AI-seg model may lead to some performance improvement compared to utilizing a single AI-seg model. However, the application of only MeshCNN with the AI-seg model demonstrated similar or relatively better performance enhancement.

### 3.4. Computational Complexity Analysis

Comparing the computational complexity of the ASHSC algorithm between five different testing environments in clinical settings, the operation time was heavily influenced by hardware configurations, with higher-performance environments yielding significantly reduced processing times (Table 3). For the surveillance stage with the MeshCNN model, in environments with the same number of CPU cores, it was observed that higher CPU grades or generations correlated with improved processing speeds. However, when comparing Test PC #3 and Test PC #4, it is evident that simply increasing the number of cores did not necessarily lead to enhanced computational efficiency. A comparative analysis of Test PC #2, Test PC #3, and Test PC #4 reveals that the operational time of the MeshCNN model was predominantly influenced by CPU processing rather than GPU processing. For the correction stage with the GenAI model, performance of GPU processing is the major factor for the operating speed. As GPU performance improves, the execution time decreases. Based on the findings of this experiment, the recommended GPU specification for utilizing the GenAI-based correction stage was identified to be at least equivalent to the RTX 2080 Super. The operation time of the ASHSC algorithm on the minimum specification PC (Test PC #2) used for executing AI-SW within a clinical environment was recorded at 97 s. The maximum operational time of the recorded ASHSC algorithm was approximately 305 s on the documentation PC (Test PC #1) in the clinical setting. The minimum driving time of the ASHSC algorithm was about 66.3 s measured on the AI development PC (Test PC #5).

### 3.5. Potential Impact of Unnecessary Corrections

**Figure 6:**
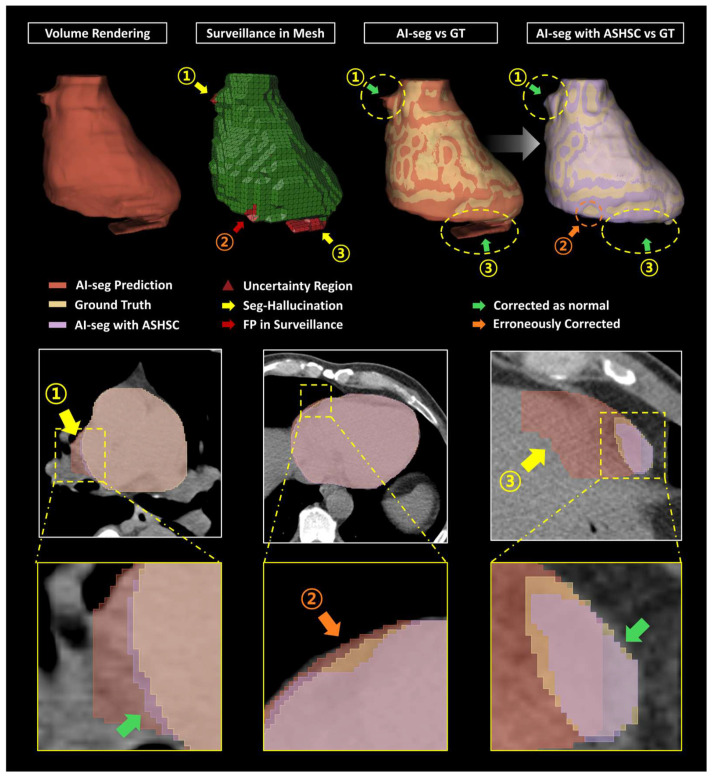
Examples of appropriate corrections and unnecessary corrections performed by the ASHSC algorithm. The yellow arrow indicates the Seg-Hallucination, the red arrow signifies the false positives in the surveillance stage, the green arrow denotes corrected regions deemed as normal, and the orange arrow highlights erroneously corrected regions.

In the quantitative performance evaluation of the ASHSC algorithm, it was ascertained that the uncertainty regions (① and ③ in Figure 6) effectively monitored during the surveillance stage led to a substantial reduction in the Seg-Hallucination areas, thus aligning them more closely with the ground truth (GT). Nevertheless, the organ mask reconstructions generated through the 3D-CycleGAN exhibited good consistencies, occasionally presenting over- or under-segmentation results, particularly at certain localized boundary regions (green arrows in Figure 6). Furthermore, among the datasets designated for on-demand correction (SQ-level < 95.3%), some instances captured false-positive uncertainty regions (② in Figure 6). Below the SQ-level threshold, these FP regions in the surveillance stage underwent a size-based filtering process prior to engaging in the GenAI-based correction stage; however, lossless restitution of these areas remains elusive for the current algorithm in its proof-of-concept version. This unnecessary correction serves as one of the critical determinants for a diminished concordance observed between the ultimately reconstructed results and the GT. In other words, unnecessary corrections imply a significant potential to diminish the analytical precision of quantitative imaging biomarkers.

## 4. Discussion

In this study, we novelly propose the proof-of-concept algorithm making use of mesh features for surveilling the Seg-Hallucination and produced a synergistic effect via GenAI-based on-demand automatic correction. To achieve this goal, we developed a fully automated two-stage approach: one is the surveillance stage with the MeshCNN model, which aims to detect the Seg-Hallucination with quantifying uncertainty regions; the other is an on-demand correction stage with the CycleGAN model, which restores the ideal anatomical organ style for refining the residual uncertainty regions.

Our test experiments on the TCIA CT dataset demonstrated highly promising performance of the proposed algorithm in both the surveillance and correction of the Seg-Hallucination. The SQ-level-based surveillance performance (AUROC = 0.94 ± 0.01) highlights the algorithm’s potential for accurately identifying erroneous segmentation results at risk of wrong quantification. The GenAI-based correction stage further improved the performance of the proposed algorithm by automatically refining the Seg-Hallucinations remaining following the MeshCNN-based surveillance stage. It indicates that the combined use of the MeshCNN and the GenAI can effectively minimize the uncertainty regions associated with Seg-Hallucination through synergistic effects.

Enhancing the algorithm’s efficiency is paramount for its real-time applicability; as medical professionals increasingly rely on timely decision making supported by AI technologies, any delay in processing can directly impact patient outcomes. When comparing the computational complexity of the ASHSC algorithm across five different PC configurations within a clinical environment, it was found that, apart from the document processing PC, execution times ranged from 66 to 97 s on PCs meeting or exceeding the specifications required for AI software operation. The results not only align with the operational demands of clinical settings but also facilitate the transition toward more responsive healthcare solutions. Continued optimization of the algorithm could maximize its operational speed, ultimately improving its integration into diverse clinical workflows.

There is still very little work for automatic surveillance of the Seg-Hallucination in a 3D perspective without the ground truth. Robinson et al. suggested direct Dice score prediction on a 2D image segmentation pair using a simple CNN regression network [30]. Zhou et al. used difference-image between the input 2D image and the newly reconstructed 2D image on predicting the Dice score using the quality regression network [31]. Huang et al. demonstrated segmentation quality with intersection over union (IOU) score by harmonizing three types of 2D-CNN models [32]. However, all these direct score regressions or extraction methods were unable to intuitively identify which part is incorrectly segmented, and both medical image data and corresponding segmented binary masks are required in the quality assessment procedure. In addition, they would suffer from robustness problems if the input images were shifted over training dataset distribution of the regression network. In contrast, our 3D MeshCNN-based method efficiently extracted uncertainty features directly related to the Seg-Hallucination and assessed the SQ-level with reliable detection performance. In addition, beyond confirming the existence of the Seg-Hallucination, it localized regions with high uncertainty in a three-dimensional space.

Similarly, the number of prior studies on the automatic correction of the Seg-Hallucination remains insufficient, and the implementation in clinical practice continues to pose challenges. Zheng et al. introduced the probability atlas map based uncertainty measurement using 3D-CNN model with user interactions [33]. Benenson et al. used a hybrid approach between human-infiltrated uncertainty region detection and CNN-based automated correction in 2D input images [34]. These methodologies are limited by their reliance on the user’s domain knowledge and expertise when attempting to accurately delineate the boundaries of incorrectly segmented areas in 3D space, especially for medical imaging fields. On the other hand, Wang et al. performed uncertainty-estimation-based correction in 2D images through test-time augmentation to enhance the robustness of DL-based model predictions [35]. However, test-time augmentation required iterative computation costs, and it still required both segmentation results and paired input images. Contrasting with the limitations of prior methodologies, the on-demand CycleGAN model is distinguished by its exclusive use of binary organ masks and operates as a fully automated process. In addition, it showed synergistic results of improved performance when integrated with the MeshCNN model of the surveillance stage.

The added value of the ASHSC algorithm, in the context of technological development, includes the minimization of uncertainty and bias in AI algorithms related to segmentation, as well as its contribution to enhancing the workflow chain of AI-based automated segmentation. In addition, its added value in clinical utility lies in providing rapid and intuitive guide to uncertainty regions for automated quality audits. It will enhance the analytical accuracy of extracted imaging biomarkers and reduce the workload of clinical researchers.

The ASHSC algorithm demonstrated enhanced performance when compared to the sole application of an AI-based segmentation model. However, the study still included certain limitations. Firstly, this investigation identified uncertainty regions that could not be adequately captured by traditional segmentation evaluation metrics (e.g., volumetric Dice score) alone, confirming the necessity of evaluating uncertainty regions from multiple perspectives as more reliable indicators of assessment. Secondly, the algorithm could accurately determine whether the segmentation result is classified as ’normal’ or ’abnormal’ based on a certain threshold; however, it may struggle to reliably differentiate between two segmentations that exhibit similar SQ-levels. Third, the study evaluated the applicability of the algorithm with respect to the organ with structured shape. Given that deep learning methods adopt inference approaches based on the distribution of the training data, additional evaluation of the robustness of the ASHSC algorithm with respect to irregularly shaped objects (e.g., malignancy lesions) is warranted. As the fourth limitation, unnecessary corrections to AI segmentation results, by the false-positive in surveillance stage, can have profound implications for the validity and utility of the ASHSC algorithm in clinical environments. When the AI segmentation outputs are altered without justification, there is a risk of introducing inaccuracies that compromise the representation of anatomical structures. Such misalignments can lead to erroneous interpretations and conclusions, ultimately affecting clinical decision making. Therefore, it is necessary to establish more robust guidelines for correction processes in AI segmentation to ensure that changes are both necessary and substantiated, thereby preserving the fidelity of the results and the integrity of subsequent analyses. Fifthly, as a limitation, this study evaluated the performance of the ASHSC algorithm using the results from a single commercial AI segmentation model. Future research could consider collecting results from a broader range of state-of-the-art models over an extended period to facilitate performance comparisons among these models without relying on ground truth. Lastly, the proposed algorithm was designed to predict short-term outcomes, serving as a vital initial step in this innovative approach. However, additional research is necessary to validate the findings using a large-scale, independent dataset, thus ensuring the algorithm’s robustness and generalizability.

## 5. Conclusions

Our study represents a pioneering proof-of-concept application that integrates both surveillance and correction of the Seg-Hallucination in a 3D perspective automatic manner. We could recognize that proposed algorithm is prone to learning key features specific to assessing the segmentation quality, and its performance could be synergistically improved collaborated with the generative AI. The study presents five key contributions. Firstly, to the best of our knowledge, this is the first study on automated audit and self-correction algorithm that integrates MeshCNN with generative AI, providing a robust solution. Distinct from prior studies, by surveilling deformities in 3D surface topology, the ASHSC algorithm innovatively eliminates a dependency of the ground truth and offers clinicians an intuitive guide for identifying uncertainty regions. Furthermore, we established a new metric, the segmentation quality level (SQ-level), which assesses the ratio of uncertainty faces on a 3D mesh surface. Even in correcting the Seg-Hallucination, we effectively minimized unnecessary correction tasks through the SQ-level-based on-demand strategy. Finally, by utilizing binary masks exclusively for surveilling and correcting the Seg-Hallucination, the ASHSC algorithm maintains manageable computational complexity, thus ensuring practical applicability across various testing environments in hospital settings. These contributions collectively not only improve the efficiency and reliability of deep-learning-based segmentation workflows but also facilitate broader implementation in clinical applications.

## Figures and Tables

**Table 1:** SQ-level-based surveillance stage performance based on three different blinded observers.

Metrics for Performance Evaluation	Validation Dataset (n = 70, Mean SQ-Level (%) = 95.2 ± 8)				Test Dataset (n = 274, Mean SQ-Level (%) = 93.8 ± 5)			
	GT by Observer 1	GT by Observer 2	GT by Observer 3	Average Score (Mean ± SD)	GT by Observer 1	GT by Observer 2	GT by Observer 3	Average Score (Mean ± SD)
SQ-level (%) threshold	97.6	97.5	97.6	97.6 ± 0.06	95.2	95.4	95.2	95.3 ± 0.12
AUROC	0.93	0.95	0.92	0.93 ± 0.02	0.93	0.94	0.94	0.94 ± 0.01
Sensitivity	0.84	0.90	0.82	0.85 ± 0.04	0.80	0.81	0.85	0.82 ± 0.03
Specificity	0.85	0.85	0.86	0.85 ± 0.01	0.91	0.90	0.90	0.90 ± 0.01
PPV	0.81	0.81	0.84	0.82 ± 0.02	0.93	0.92	0.91	0.92 ± 0.01

**Table 2:** Performance comparisons between different problem-solving methodologies.

Metrics for Performance Evaluation	Validation Dataset (n = 70, Mean SQ-Level (%) = 95.2 ± 8)				Test Dataset (n = 274, Mean SQ-Level (%) = 93.8 ± 5)			
	AI-Seg (Mean ± SD)	AI-Seg + MeshCNN (Mean ± SD)	AI-Seg + GenAI (Mean ± SD)	AI-Seg + MeshCNN + GenAI (Mean ± SD)	AI-Seg (Mean ± SD)	AI-Seg + MeshCNN (Mean ± SD)	AI-Seg + GenAI (Mean ± SD)	AI-Seg + MeshCNN + GenAI (Mean ± SD)
Volumetric Dice score	0.98 ± 0.03	0.99 ± 0.03	0.98 ± 0.03	0.99 ± 0.03	0.97 ± 0.03	0.98 ± 0.02	0.96 ± 0.03	0.98 ± 0.02
Volume error Percentage (%)	2.8 ± 8.4	1.6 ± 5.3	1.7 ± 7.2	1.4 ± 4.6	6.1 ± 7.4	3.5 ± 5.7	4.5 ± 6.6	3.4 ± 5.5
Average surface Distance (mm)	1.1 ± 3.0	0.6 ± 1.9	1.0 ± 2.5	0.5 ± 1.7	2.2 ± 3.0	1.2 ± 1.5	2.0 ± 2.3	1.1 ± 1.4
Hausdorff Distance (mm)	17.6 ± 19.4	13.9 ± 16.1	14.2 ± 18.8	9.0 ± 13.1	23.1 ± 19.3	18.0 ± 15.3	20.1 ± 17.4	13.7 ± 9.4

**Table 3:** Computational complexity comparison between different testing environments in clinical settings.

Category	Operation Time (Sec, Mean ± Std) Measurement for Test Dataset (n = 274)				
	Test PC #1 OS: Windows 11 CPU: i5-8400k (6 cores) GPU: GTX 1050 PyTorch: v2.5.1 CUDA: v12.4	Test PC #2 OS: Windows 11 CPU: i7-9700 (8 cores) GPU: RTX 2080 Su PyTorch: v2.5.1 CUDA: v12.4	Test PC #3 OS: Windows 11 CPU: i9-9900k (8 cores) GPU: RTX 2080 Su PyTorch: v2.5.1 CUDA: v12.4	Test PC #4 OS: Windows 11 CPU: R9 5950X (16 cores) GPU: RTX 3090 PyTorch: v2.5.1 CUDA: v12.4	Test PC #5 OS: Windows 11 CPU: i9-12900kf (16 cores) GPU: RTX 3090 Ti PyTorch: v2.5.1 CUDA: v12.4
Usage purpose	Documentation	Clinical AI-SW	Clinical AI-SW	AI development	AI development
Facilitating cost	Low	Moderate	Medium	High	Very High
Surveillance stage (MeshCNN)	84.3 ± 12.3	74.9 ± 10.7	65.2 ± 9.0	66.9 ± 9.6	49.7 ± 7.0
Correction stage (GenAI)	220.7 ± 19.0	22.2 ± 2.0	21.2 ± 1.8	18.2 ± 1.5	16.6 ± 1.4
ASHSC algorithm	305.0 ± 20.5	97.0 ± 10.5	86.4 ± 8.8	85.2 ± 9.4	66.3 ± 6.9

## Data Availability

The datasets generated or analyzed during the study are available from the corresponding author upon reasonable request.
